# Breeding for durable resistance against biotrophic fungal pathogens using transgenes from wheat

**DOI:** 10.1007/s11032-024-01451-2

**Published:** 2024-01-22

**Authors:** Marcela Camenzind, Teresa Koller, Cygni Armbruster, Esther Jung, Susanne Brunner, Gerhard Herren, Beat Keller

**Affiliations:** 1 Department of Plant and Microbial Biology, University of Zurich, Zollikerstrasse 107, 8008 Zurich, Switzerland; 2 Agroscope, Reckenholzstrasse 191, 8046 Zurich, Switzerland

**Keywords:** Transgenic crops, Disease resistance, Field trials, Wheat, Barley, Fungal pathogens

## Abstract

Breeding for resistant crops is a sustainable way to control disease and relies on the introduction of novel resistance genes. Here, we tested three strategies on how to use transgenes from wheat to achieve durable resistance against fungal pathogens in the field. First, we tested the highly effective, overexpressed single transgene *Pm3e* in the background of spring wheat cultivar Bobwhite in a long-term field trial over many years. Together with previous results, this revealed that transgenic wheat line Pm3e#2 conferred complete powdery mildew resistance during a total of nine field seasons without a negative impact on yield. Furthermore, overexpressed *Pm3e* provided resistance to powdery mildew isolates from our worldwide collection when crossed into the elite wheat cultivar Fiorina. Second, we pyramided the four overexpressed transgenes *Pm3a*, *Pm3b*, *Pm3d*, and *Pm3f* in the background of cultivar Bobwhite and showed that the pyramided line Pm3a,b,d,f was completely resistant to powdery mildew in five field seasons. Third, we performed field trials with three barley lines expressing adult plant resistance gene *Lr34* from wheat during three field seasons. Line GLP8 expressed *Lr34* under control of the pathogen-inducible *Hv-Ger4c* promoter and provided partial barley powdery mildew and leaf rust resistance in the field with small, negative effects on yield components which might need compensatory breeding. Overall, our study demonstrates and discusses three successful strategies for achieving fungal disease resistance of wheat and barley in the field using transgenes from wheat. These strategies might confer long-term resistance if applied in a sustainable way.

**Supplementary Information:**

The online version contains supplementary material available at 10.1007/s11032-024-01451-2.

## Introduction

Crop production is threatened by numerous pathogens leading to disease and yield losses in agriculture. Two approaches are commonly employed to combat diseases: the use of pesticides and the use of resistant crop cultivars. Breeding for resistant crops is the more sustainable approach and often relies on the identification and introduction of new resistance genes from the crop’s breeding pool. *Pm3* is a major resistance gene conferring resistance to powdery mildew (*Blumeria graminis* f.sp. *tritici*, *Bgt*) in wheat. To date, there are 17 identified *Pm3* alleles which provide resistance to different spectra of *Bgt* isolates (Yahiaoui et al. 2004, 2006, 2009; Srichumpa et al. 2005; Bhullar et al. 2009, 2010). They encode nucleotide-binding domain, leucine-rich repeat receptor proteins (NLRs) which confer race-specific resistance upon recognition of specific *Bgt* avirulence proteins (Avrs). To study and compare field resistance conferred by individual *Pm3* alleles, transgenic wheat lines carrying single overexpressed *Pm3* alleles were earlier created by biolistic transformation of spring wheat cultivar Bobwhite (Brunner et al. 2011, 2012; Koller et al. 2019). The allele, *Pm3e* specifically, was shown to confer strong powdery mildew resistance without any fitness cost, making it a potentially valuable allele for wheat breeding (Koller et al. 2019). However, race-specific resistance genes such as NLRs are prone to breakdown due to homogenous, large-scale deployment driving strong selection for gain-of-virulence pathogen mutations (Papaïx et al. 2018). An approach for improving resistance durability, as well as broadening the resistance spectrum, is the pyramiding of multiple resistance genes in a crop cultivar (Koller et al. 2018): There, for a successful infection, the pathogen must adapt multiple avirulence effectors to avoid recognition by the different resistance gene products. Pyramiding can be achieved using classical crossbreeding supported by marker-assisted approaches as was shown for the pyramiding of different *Magnaporthe oryzae* resistance genes in rice (Khan et al. 2018; Wu et al. 2019). However, pyramiding can also be achieved faster and more efficiently using genetic transformation approaches. In potato cultivar Desiree, for example, three late blight resistance genes were stacked using *Agrobacterium tumefaciens*-mediated transformation and the pyramided lines showed improved resistance compared to plants with single resistance genes during two field trials (Haesaert et al. 2015). Koller et al. (2018) described another example where transgenic wheat lines with two pyramided *Pm3* alleles, generated by biolistic transformation and crossbreeding, showed enhanced powdery mildew resistance in the field compared to single transgenic lines. The improved resistance was attributed to additive transgene expression levels. Pyramiding of alleles of the same gene in a genetically stable way, as done for *Pm3*, can currently only be achieved by transformation and not by conventional crossing because the alleles are in the same genetic locus. Another class of resistance genes are quantitative, partially acting resistance genes. They are also referred to as adult plant resistance (*APR*) genes since resistance is only functional in the adult plant stage and not at the seedling stage as is the case for major resistance genes. Unlike major, race-specific resistance genes, *APR* genes do not completely abolish disease, rather they provide partial resistance which is characterized by reduced fungal growth. Consequently, they exert lower selection pressure on the pathogen population to adapt and overcome resistance and are generally considered to be more durable (Dinglasan et al. 2022). *APR* genes mostly confer partial and durable resistance to all races of a pathogen and in a few cases even to multiple pathogens (Kou and Wang 2010). Three genes conferring such broad-spectrum, multi-pathogen resistance were found in wheat. *Lr34* (= *Yr18/Sr57/Pm38*), *Lr46* (= *Yr29/Sr58/Pm39*), and *Lr67* (= *Yr46/Sr55/Pm46*) confer partial resistance against all races of the fungal pathogens causing leaf rust (*Puccinia triticina*), stripe rust (*Puccinia striiformis* f.sp. *tritici*), stem rust (*Puccinia graminis* f.sp. *tritici*), and powdery mildew (*Blumeria graminis* f.sp. *tritici*) (Ellis et al. 2014; Krattinger et al. 2019). These genes are associated with leaf tip necrosis (LTN), a senescence-like phenotype in the flag leaf of adult plants (Singh 1992; Krattinger et al. 2016). *Lr34* has become one of the most frequently used disease-resistance genes in wheat breeding and despite its widespread use has remained effective for over 100 years (Kolmer et al. 2008; Krattinger et al. 2013). Two predominant *Lr34* alleles are found in wheat, but resistance is provided by only one (*Lr34res*), which evolved from the ancestral susceptible allele (*Lr34sus*) by two gain-of-function mutations (Krattinger et al. 2013). *Lr34* encodes an ATP-binding cassette (ABC) transporter protein shown to mediate the transport of the phytohormone abscisic acid (ABA), which accumulates at the leaf tip (Krattinger et al. 2009, 2019; Bräunlich et al. 2021). However, the exact mechanisms of how *Lr34* provides resistance remain elusive. *Lr34* has been successfully transferred to most major cereals such as barley, rice, maize, durum wheat, and sorghum (Risk et al. 2013; Boni et al. 2018; Krattinger et al. 2016; Sucher et al. 2017; Rinaldo et al. 2017; Schnippenkoetter et al. 2017), indicating that the molecular mechanism for resistance must be conserved in all these plant species. In barley, *Lr34* expression led to disease resistance already at the seedling stage, however, accompanied by premature LTN and reduced plant fitness (Risk et al. 2013; Chauhan et al. 2015). These negative effects were reduced without compromising resistance by using a pathogen-inducible promoter which decreased *Lr34* expression levels in transgenic barley lines (Boni et al. 2018). In this study, we tested three approaches for improving field resistance to various biotrophic fungal pathogens using transgenes from wheat. First, we completed a long-term field study for powdery mildew resistance conferred by the overexpressed transgene *Pm3e*. Second, we tested pyramided line Pm3a,b,d,f overexpressing four transgenes during five field seasons. Third, we tested three barley lines expressing the adult plant resistance gene *Lr34* from wheat during three field seasons for resistance to biotrophic fungal pathogens.

## Materials and methods

### Plant material

The wheat single transgenic lines Pm3a, Pm3b, Pm3d, and Pm3f in the background of spring wheat cultivar Bobwhite SH 98 26 were previously generated by Brunner et al. (2011, 2012) by biolistic transformation (named Pm3a#1, Pm3b#1, Pm3d#1, Pm3f#1). Lines Pm3a,d and Pm3b,f were previously generated and described by Stirnweis et al. (2014). Pyramided line Pm3a,b,d,f was obtained by crossing parental lines Pm3a,d and Pm3b,f, followed by several rounds of self-fertilization. The T_5_ generation was then analyzed by PCR for the presence of the four alleles *Pm3a*, *Pm3b*, *Pm3d*, and *Pm3f* using allele-specific primers. *Pm3a* primers: 5′-TGTATCATCTGGAACCAGCGT-3′ (forward); 5′-CCATAGTTGGATCAACTTCGCTA-3′ (reverse). *Pm3b* primers: 5′-TTTAGCCCTGCCTTCATACG-3′ (f); 5′-AGTAGCTCGGGAATCTTTCCA-3′ (r). *Pm3d* primers: 5′-GAATCCCTTTGGCTTGAAAGA-3′ (f); 5′-GCATAATCTGGAACATCGTATGG-3′ (r). *Pm3f* primers: 5′-CGGGTATTATTCCAGCACATGT-3′ (f); 5′-GAGTAGAAATGATTCTGTGCCTCTA-3′ (r). Based on this, the pyramided line homozygous for all four *Pm3* alleles was selected. Transgenic Bobwhite line Pm3e#2 was previously generated and described by Koller et al. (2019). *Pm3e*-Fiorina BC3F2 seedlings were obtained by crossing Pm3e#2 and elite spring wheat cultivar Fiorina, followed by three back-crosses with Fiorina and subsequent selfing. Genotyping and anti-HA western blots confirmed the presence and expression of *Pm3e-HA* in each generation. The transgenic barley lines used in this study were BG9 generated and described in Risk et al. (2013), as well as GLP8 and GLP11 generated and described in Boni et al. (2018). In BG9, *Lr34* expression is controlled by the native wheat promoter, while in GLP8 and GLP11, *Lr34* expression is controlled by the pathogen-inducible promoter *Hv-Ger4c*. For seedling infection tests, the plants were grown in the greenhouse under long-day conditions (20/16 °C, 16 h photoperiod, 60% humidity).

### Field trial set-up and disease scoring

Legal permits for field experiments involving genetically modified plants were obtained by the Federal Office for the Environment prior to the field trials (permits B18001, B18004, and B20002) under the Release Ordinance 2008 and the Gene Technology Act 2003 in compliance with the EU Directive 2001/18/EC. The field trials were performed during the five field seasons 2019, 2020, 2021, 2022, and 2023, essentially as described in Koller et al. (2019). Wheat and barley lines for resistance tests were grown in test plots of 1.5 m × 1.0 m at the so-called “Protected Site” (www.protectedsite.ch), an experimental field site for research trials with transgenic crops located at Agroscope in Zurich Reckenholz (Brunner et al. 2021; Romeis et al. 2013). Four test plots per genotype were grown in a randomized complete block design. Wheat powdery mildew test plots were flanked by infection rows consisting of the powdery mildew susceptible wheat breeding line FAL94632 and cultivar Kanzler. Pots with susceptible wheat plants pre-infected in the greenhouse with Swiss powdery mildew isolate *Bgt* 96224 (Wicker et al. 2013) were planted into the infection rows as described by Koller et al. (2019). Barley powdery mildew test plots were flanked by infection rows consisting of the powdery mildew susceptible barley cultivar Golden Promise inoculated with *Blumeria graminis* f.sp. *hordei* (*Bgh*) isolate K1 (Boni et al. 2018). Barley leaf rust test plots were artificially infected with *Puccinia hordei* isolate 1.2.1 (Risk et al. 2013). Scoring of powdery mildew and flowering was performed as described by Brunner et al. (2011, 2012). For leaf rust scoring, the percentage of flag leaf area covered with rust pustules was estimated approximately every 3 days after the onset of the disease. Yield plots were sown using a 7-row plot drill with 0.18-m row spacing and had a size of 6.5 m × 1.5 m. They were fungicide-treated. A sowing error in field season 2023 led to some replicate yield plots with less than the standard seven sowing rows. However, the number of seed sowed per plot was the same. Yield and 1000-grain weight were measured by five independent yield plot replicates per line and year.

### Transgene expression analysis

Flag leaf samples (wheat) and penultimate leaf samples (barley) were collected from field-grown plants each year. For each of the four biological replicates (= four plots), three plant samples were pooled and frozen immediately in dry ice. RNA extraction was performed as described in Bräunlich et al. (2021) using the Dynabeads® mRNA DIRECT™ Purification Kit (Thermo Fisher Scientific, Waltham, MA, USA). RNA (3 µL) was reverse transcribed in a total volume of 10 µL using the Maxima H Minus First Strand cDNA Synthesis Kit with dsDNase (Thermo Fisher Scientific). Quantitative reverse transcription PCR (RT-qPCR) experiments were performed according to MIQE guidelines (Bustin et al. 2009) using the KAPA SYBR® FAST qPCR Master Mix (Kapa Biosystems) on a CFX96 Real-Time PCR Detection System (Bio-Rad, Hercules, CA, USA). Reactions were run in technical triplicates and in 10 µL reaction volume using 4 µL of 1:10 diluted cDNA and 250 nM primers. For total *Pm3* expression analysis in wheat, the following primers were used: 5′-CTGGAGTGTCTGTCGGGAGAG-3′ (forward) and 5′-GCATCTAGCCATTTGCGTTTG-3′ (reverse). These *Pm3* primers did not distinguish between the four *Pm3* alleles *Pm3a*, *Pm3b*, *Pm3d*, and *Pm3f* (Yahiaoui et al. 2004, 2006) since it was not possible to design reliable allele-specific qPCR primers due to high sequence similarity between them. Allele-specific primers previously designed for genotyping were tested for qPCR; however, they were not suitable. mRNA expression levels were normalized to reference gene Ta.6863 (Hurni et al. 2013; Koller et al. 2018, 2019). For *Lr34* expression analysis in barley, the following primers were used: 5′-GACAGCGCCAGAATGGTGC-3′ (forward) and 5′-GACATCAACCCTGTCAATTC-3′ (reverse). ADP ribosylation factor (*ADPRF*) was used as a reference gene (Giménez et al. 2011). Gene expression data was analyzed using the CFX Maestro software version 2.2 (Bio-Rad).

### Pm3-HA protein detection

Total protein was extracted from field-grown wheat flag leaf samples (three pooled leaves per plot) in protein extraction buffer containing 15 mM NaCl, 5 mM Tris–HCl pH 7.5, 0.5% Triton X-100 and one tablet cOmplete™ EDTA-free protease inhibitor cocktail (Roche). The total protein concentration of the extract was determined using the Pierce BCA Protein Assay Kit (Thermo Fisher Scientific) according to the manufacturer’s protocol. Equal amounts of total protein were loaded and separated on 8% SDS polyacrylamide gels and transferred to a PVDF membrane. Anti-HA-HRP antibody (rat monoclonal, clone 3F10, Roche) was used in a 1:1000 dilution for detection of Pm3-HA, together with WesternBright EC HRP substrate (Advansta, San Jose, CA, USA). Chemiluminescence was captured using the Fusion FX Imaging System (Vilber Lourmat, Eberhardzell, Germany).

### Powdery mildew infection tests at the seedling stage

Virulence phenotyping experiments at the seedling stage were performed by placing 3-cm-long first leaf segments of approximately 12-day-old wheat plants with the adaxial side up on 0.5% agar plates supplemented with 0.24 mM benzimidazole (Parlange et al. 2011). Leaf segments were infected with *Bgt* spores by homogenous spraying with a single-use glass pipette and incubated at 20 °C for 7 days with 16 h of light per day to allow for sufficient colony growth. Virulence scoring was performed 7 days after infection by estimating the percentage of leaf coverage with fungal colonies (LC). The virulence phenotype was then categorized into three classes: virulent for LC = 70–100%, intermediate for LC = 10–70%, and avirulent for LC > 10%. The powdery mildew-susceptible cultivar Kanzler was included as a control.

### Statistical analyses

Data were presented as single data points and boxplots showing the median, the first, and the third quartile. Analysis of variance (ANOVA) followed by Tukey’s honestly significant difference (HSD) test was performed to evaluate statistical differences between groups using the “agricolae” package for RStudio version 4.1.3 (de Mendiburu 2021). Student’s *t*-tests were also carried out in RStudio. The significance threshold alpha = 0.05 was used for all analyses.

## Results

### Overexpressed *Pm3e* conferred strong powdery mildew field resistance during nine field seasons without negative impact on yield

**Fig. 1:**
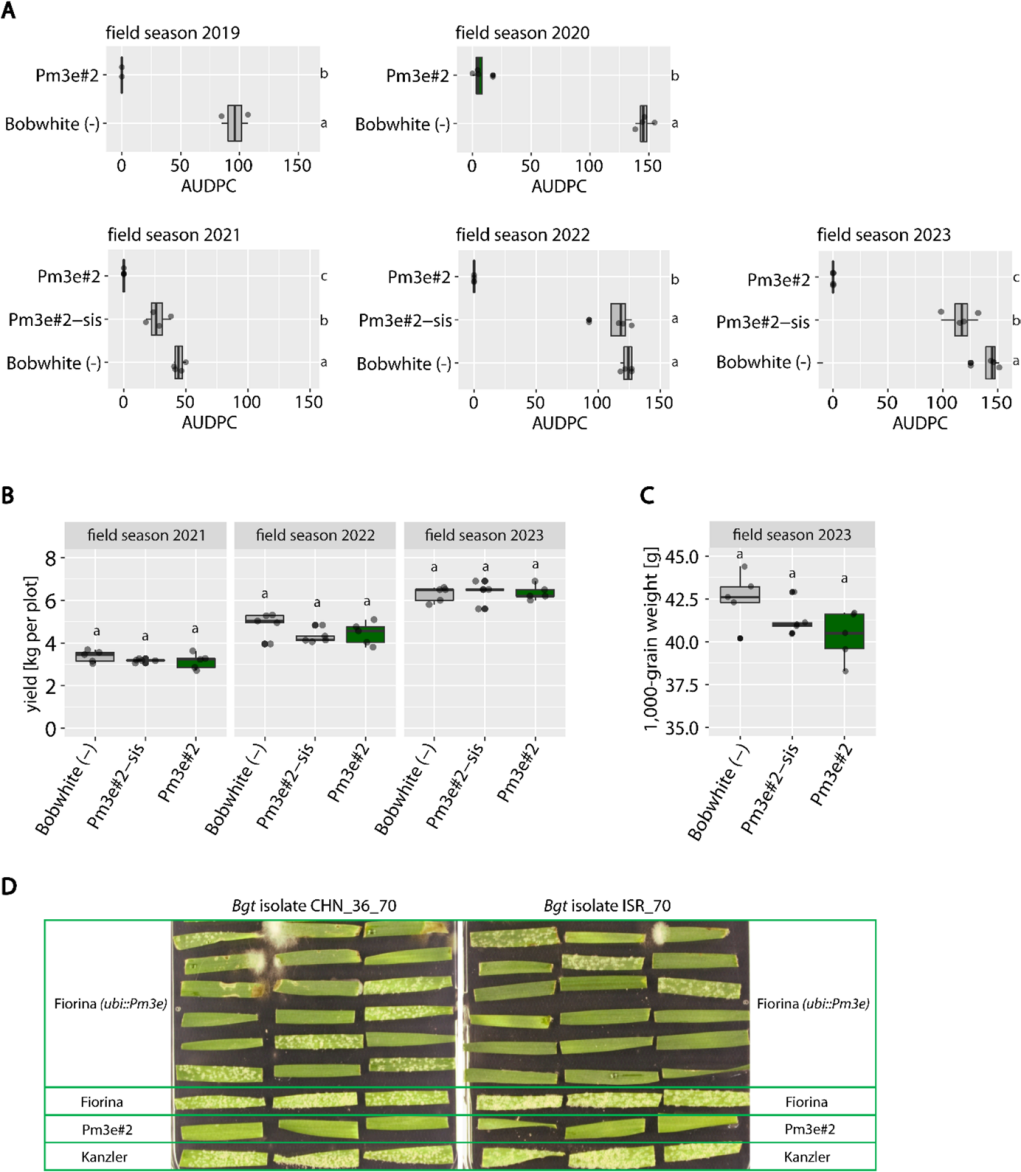
Transgenic *Pm3e* confers strong powdery mildew resistance in the field. **A** Powdery mildew infection of field-grown *Pm3e*-transgenic wheat. The area under disease progress curve (AUDPC) values of four independent plots per genotype (except 2019: two plots per genotype) are shown. Differing letters indicate a significant difference in infection calculated by Tukey’s honestly significant difference test (Tukey HSD test, *α* = 0.050). Plot yield **B** and 1000-grain weight of field-grown Pm3e#2 **C**, its corresponding sister line Pm3e#-sis and untransformed Bobwhite. Yield and 1000-grain were measured from five independent, fungicide-treated plots per genotype. The letters above bars indicate a significant difference in yield or 1000-grain weight as determined by the Tukey HSD test (*α* = 0.050). **D** Representative pictures of *Blumeria graminis* f.sp. *tritici* (*Bgt*) infection tests with Fiorina (*ubi::Pm3e*) BC3F2 plants using two different *Bgt* isolates virulent on Fiorina and avirulent on Pm3e#2. For each isolate, 18 individual seedlings of Fiorina (*ubi::Pm3e*) BC3F2 were scored, and three biological replicates were scored for Fiorina, Pm3e#2, and Kanzler. As expected, there was segregation for the *Pm3e* transgene in the backcross genotypes. The susceptible wheat cultivar Kanzler was included as a positive control

We tested transgenic Bobwhite event Pm3e#2, which overexpresses C-terminally HA-tagged *Pm3e* under the maize *ubi* promoter (Koller et al. 2019), during the five field seasons 2019 to 2023 for powdery mildew resistance. During the four field seasons from 2020 to 2023, the infection rows planted next to the test plots were artificially infected with Swiss powdery mildew isolate *Bgt* 96224 to ensure an evenly distributed powdery mildew infection in the field trial. In field season 2019, the powdery mildew infection originated entirely from the naturally occurring local powdery mildew population, which reliably causes powdery mildew disease every year at the field trial site in Zürich Reckenholz, Switzerland. Pm3e#2 showed strong powdery mildew resistance in all five field seasons 2019, 2020, 2021, 2022, and 2023 tested in this study (Fig. 1A). Together with our previous studies performed during field seasons 2015, 2016, 2017, and 2018 published in Koller et al. (2019), this resulted in a long-term study of nine field seasons in which Pm3e#2 showed strong powdery mildew resistance. In the four field seasons of 2019, 2021, 2022, and 2023 tested in this study, Pm3e#2 had AUDPC values = 0. The small powdery mildew infection in field season 2020 (AUDPC value average = 7) possibly originates from some seed admixtures during sowing (seed carryover from neighboring plots). In contrast, non-transformed Bobwhite and sister line Pm3e#2-sis (null segregant of Pm3e#2) were both powdery mildew susceptible in all tested field seasons (Pm3e#2-sis was only tested during field seasons 2021, 2022, and 2023) with AUDPC values up to 150 and 125, respectively (Fig. 1A). During the field seasons 2021 to 2023, we additionally tested if overexpressed *Pm3e* in transgenic event Pm3e#2 had a negative effect on yield compared to sister line Pm3e#2-sis and non-transformed Bobwhite. To test the yield potential in the absence of powdery mildew infection, we treated all yield plots with fungicides. In all three field seasons, there was no statistically significant difference in yield between Pm3e#2, Pm3e#2-sis, and non-transformed Bobwhite (Fig. 1B). However, the average amount of yield varied considerably between the different field seasons with an average of 3.2 kg per plot in 2021, 4.5 kg per plot in 2022, and 6.4 kg per plot in 2023. This could be explained by the prevailing weather conditions during the different field seasons, which can influence flowering time and duration of the grain-filling period, thereby influencing yield (Akter and Rafiqul Islam 2017; Yadav and Ellis 2017). In field season 2021, weather was generally unfavorable for wheat growth and wheat lines flowered later in June compared to the other field seasons (Suppl. Fig. S1A), likely leading to a reduced grain-filling period and generally lower yields. In addition to the yield data, we measured the 1000-grain weight of Pm3e#2, Pm3e#2-sis, and Bobwhite in field season 2023 and found no significant difference between the three lines (Fig. 1C).

### *Pm3e* conferred powdery mildew resistance in elite wheat cultivar Fiorina

Furthermore, we sought to determine whether overexpressed *Pm3e* could be used to improve elite wheat cultivars with only intermediate powdery mildew resistance such as the spring wheat cultivar Fiorina (Strebel et al. 2022). We therefore performed a cross between Pm3e#2 and Fiorina, followed by three back-crosses with Fiorina, and tested powdery mildew resistance of the resulting, individual BC3F2 plants in seedling infection assays. We used *Bgt* isolates from our group’s worldwide *Bgt* collection and selected one *Bgt* isolate from Switzerland (*Bgt* CHE_96224), two from China (*Bgt* CHN_36_3 and *Bgt* CHN_36_70), and two from Israel (*Bgt* ISR_70 and *Bgt* ISR_103) which were virulent on parental cultivar Fiorina but avirulent on parental line Pm3e#2. We performed infection tests with the five *Bgt* isolates on *Pm3e-*Fiorina BC3F2—seedlings. The seedlings segregated in the expected 3:1 ratio for powdery mildew resistance: susceptibility over all five tested *Bgt* isolates, showing the expected powdery mildew resistance effect of *Pm3e* in *Pm3e-*Fiorina (results for *Bgt* CHN_36_70 and *Bgt* IRS_70 are shown in Fig. 1D;* X*^2^ (1, *N* = 18) = 0.67, *p* = 0.41, for both isolates). Together, these results showed that overexpressed *Pm3e* provided strong powdery mildew resistance during nine field seasons without compromising yield and demonstrated its potential to improve powdery mildew resistance in elite wheat cultivars.

### Pyramiding of four overexpressed *Pm3* alleles in wheat line Pm3a,b,d,f resulted in strong powdery mildew resistance during five field seasons

**Fig. 2:**
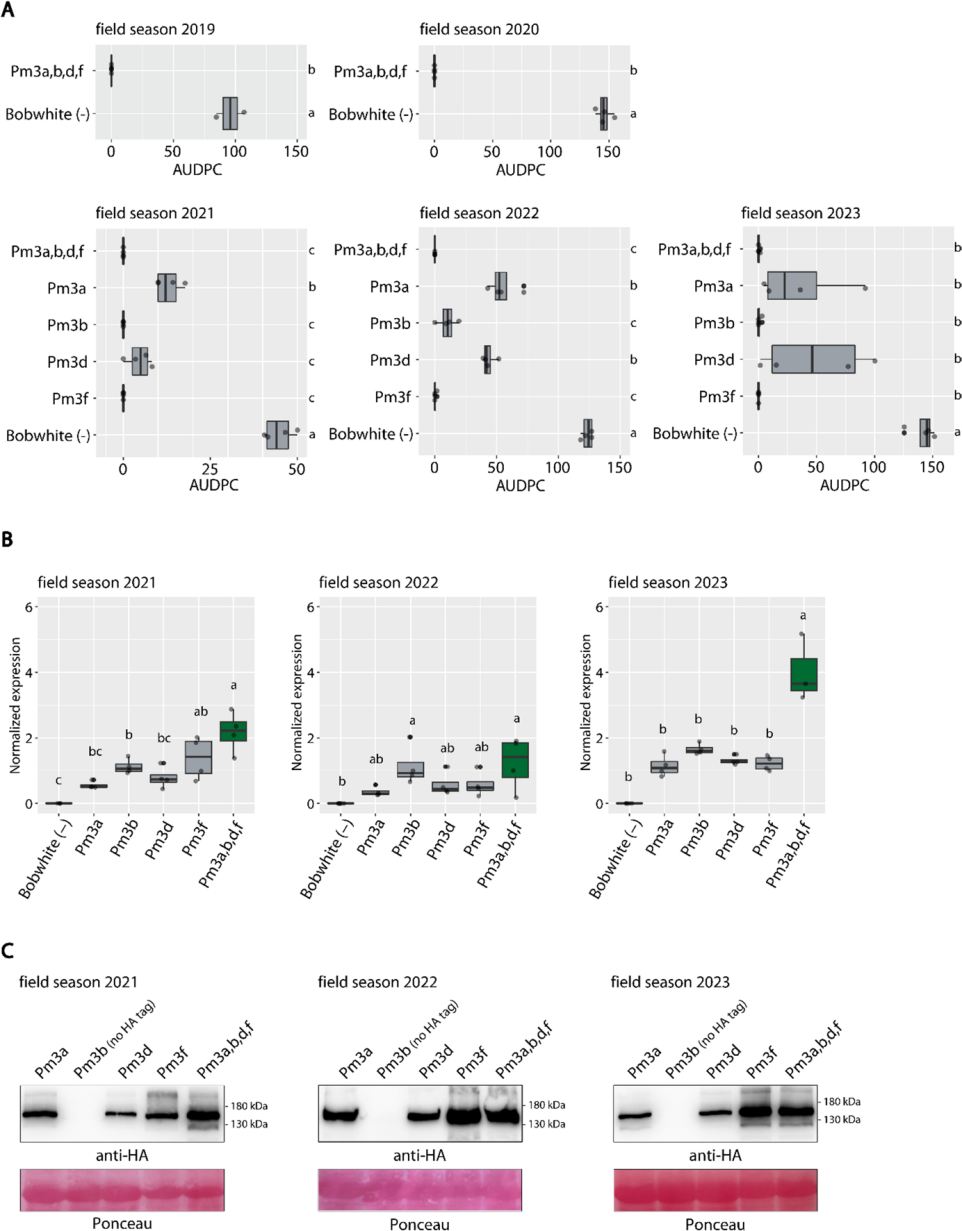
Pyramided wheat line Pm3a,b,d,f shows strong powdery mildew resistance in the field. **A** Powdery mildew infection of field-grown, *Pm3* allele-pyramided transgenic wheat. The area under disease progress curve (AUDPC) values of four independent plots per genotype (except for 2019: five plots Pm3a,b,d,f and two plots non-transformed Bobwhite) are shown. Differing letters indicate significant differences in infection (Tukey HSD test, *α* = 0.050). **B** Total *Pm3* expression relative to reference gene Ta.6863 in field-grown flag leaf samples determined by RT-qPCR. Letters above bars indicate significant differences in expression levels (Tukey HSD test, *α* = 0.050). **C** Total Pm3-HA protein accumulation in flag leaf samples from field plants. Each sample contains three pooled flag leaf segments from different plant individuals. Total protein concentration was measured and adjusted to the same concentration prior to loading. Ponceau staining indicates equal loading

We generated the transgenic pyramided wheat line Pm3a,b,d,f in the background of cultivar Bobwhite by crossing parental transgenic Bobwhite lines Pm3a,d and Pm3b,f, which carry the two overexpressed transgenes *Pm3a* and *Pm3d*, and the two overexpressed transgenes *Pm3b* and *Pm3f*, respectively. We self-fertilized the progeny of the crosses for up to five generations and selected the pyramided line which carries all four transgenes in a homozygous state. All transgenes were expressed under the maize ubiquitin (*ubi*) promoter and were C-terminally fused to the sequence encoding the HA epitope tag for protein detection, except for *Pm3b*, which has no epitope tag, i.e., ubi::*Pm3a-HA*, ubi::*Pm3b*, ubi::*Pm3d-HA*, and ubi::*Pm3f-HA*. We then tested pyramided line Pm3a,b,d,f during five field seasons for powdery mildew resistance. During all five field seasons, pyramided wheat line Pm3a,b,d,f showed complete powdery mildew resistance with AUDPC values = 0 (Fig. 2A). In contrast, the powdery mildew-susceptible, non-transformed Bobwhite had AUDPC values ranging between 50 and 150, depending on the field season’s weather-dependent disease pressure. In field seasons 2021, 2022, and 2023, we also grew the two parental lines Pm3a,d and Pm3b,f, and the four grand parental lines Pm3a, Pm3b, Pm3d, and Pm3f as additional controls. Each grand parental line carries one of the four overexpressed transgenes *Pm3a*, *Pm3b*, *Pm3d*, or *Pm3f*. Consistent with previous studies, the grand parental line Pm3f was also completely powdery mildew resistant with AUDPC values = 0 in all three field seasons (Fig. 2A). Similarly, the grand parental line Pm3b showed very strong powdery mildew resistance in all three tested field seasons, while lines Pm3a and Pm3d showed only intermediate resistance but with clearly less symptoms than susceptible Bobwhite. We collected flag leaf samples of the field-grown transgenic wheat lines to measure total *Pm3* expression and total Pm3-HA protein accumulation. RT-qPCR analysis showed higher total *Pm3* expression in line Pm3a,b,d,f compared to the grand parental lines in 2021, 2022, and 2023 (Fig. 2B). Expression data from 2023 suggests additive expression levels of the four *Pm3* alleles in pyramided line Pm3a,b,d,f; however, data from 2021 and 2022 did not confirm truly additive expression levels of the four *Pm3* alleles (Fig. 2B). Similarly inconclusive results were obtained from the protein analysis: total Pm3-HA protein accumulation data of field-grown flag leaf samples did not show any obvious additive Pm3-HA accumulation in line Pm3a,b,d,f. Surprisingly, the total amount of Pm3-HA protein accumulation in wheat line Pm3a,b,d,f was similar to the amount of Pm3f-HA protein accumulation in grand parental line Pm3f (Fig. 2C).

To evaluate possible pleiotropic effects of the overexpressed transgenes in the transgenic lines, we additionally scored flowering dates of the field-grown lines. In field season 2021, there was no significant difference in flowering time between pyramided line Pm3a,b,d,f and untransformed Bobwhite; however, in field seasons 2022 and 2023, Pm3a,b,d,f had a slight delay in the flowering of approximately 2 days, similar to Pm3f (Suppl. Fig. S1A). Chlorotic leaves were observed in field-grown grand parental line Pm3f as described previously by Brunner et al. (2012). Interestingly, in pyramided line Pm3a,b,d,f, this pleiotropic effect was significantly diminished (Suppl. Fig. S1B). Overall, these results showed that pyramiding of the four *Pm3* alleles *Pm3a*, *Pm3b*, *Pm3d*, and *Pm3f* provided complete powdery mildew resistance during five field seasons while reducing the leaf chlorosis observed in the single transgenic line Pm3f.

### Wheat transgene *Lr34* provides leaf rust and powdery mildew resistance in field-grown transgenic barley

**Fig. 3:**
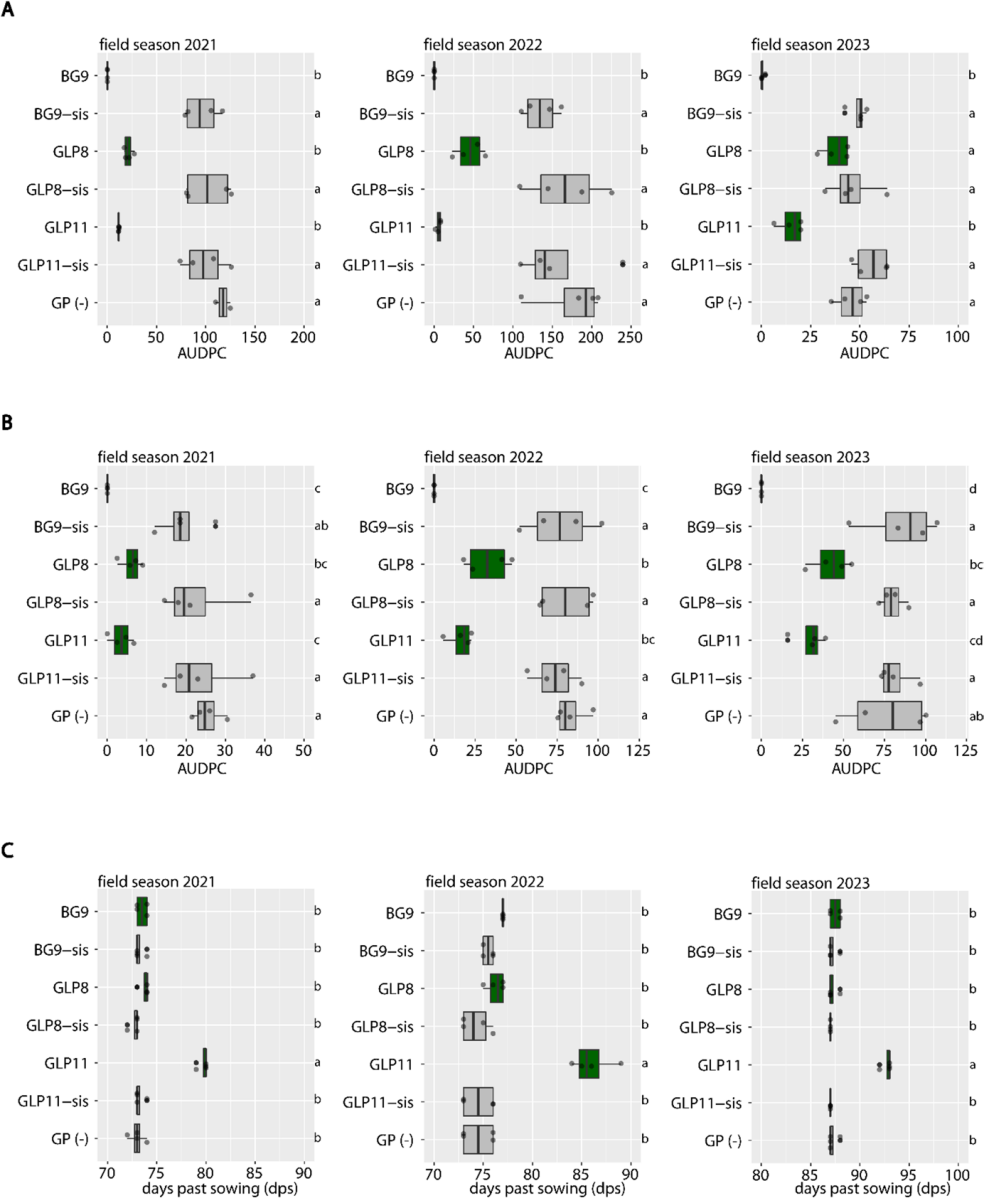
*Lr34* from wheat provides leaf rust and powdery mildew resistance in field-grown transgenic barley lines BG9, GLP8, and GLP11. Leaf rust infection (**A**) and powdery mildew infection (**B**). The area under disease progress curve (AUDPC) values of four independent plots per genotype are shown. **C** Flowering dates of field-grown barley lines. Differing letters indicate significant differences in infection or flowering time (Tukey HSD test, *α* = 0.050)

We grew three transgenic barley lines expressing the wheat transgene *Lr34* in field seasons 2021, 2022, and 2023 and tested them for barley leaf rust (*Puccinia hordei*) and barley powdery mildew (*Blumeria graminis* f. sp. *hordei* (*Bgh*)) resistance. Barley line BG9 expresses *Lr34* under the native *Lr34* promoter from wheat (Risk et al. 2013), while barley lines GLP8 and GLP11 express *Lr34* under the pathogen-inducible promoter *Hv-Ger4c*, which originates from a barley gene (Boni et al. 2018). Barley leaf rust resistance test plots were artificially infected with *P. hordei* isolate 1.2.1. In the two field seasons 2021 and 2022, all three transgenic barley lines BG9, GLP8, and GLP11 were significantly more resistant against barley leaf rust compared to their corresponding sister lines BG9-sis, GLP8-sis, GLP11-sis, and untransformed barley background cultivar Golden Promise (GP) (Fig. 3A). In field season 2023, however, GLP8 showed a similar level of rust infection as GLP8-sis and GP, possibly because differences might not have been clearly visible due to the lower disease pressure, reflected by the reduced overall AUDPC values in 2023 compared to 2022 and 2021.

**Fig. 4:**
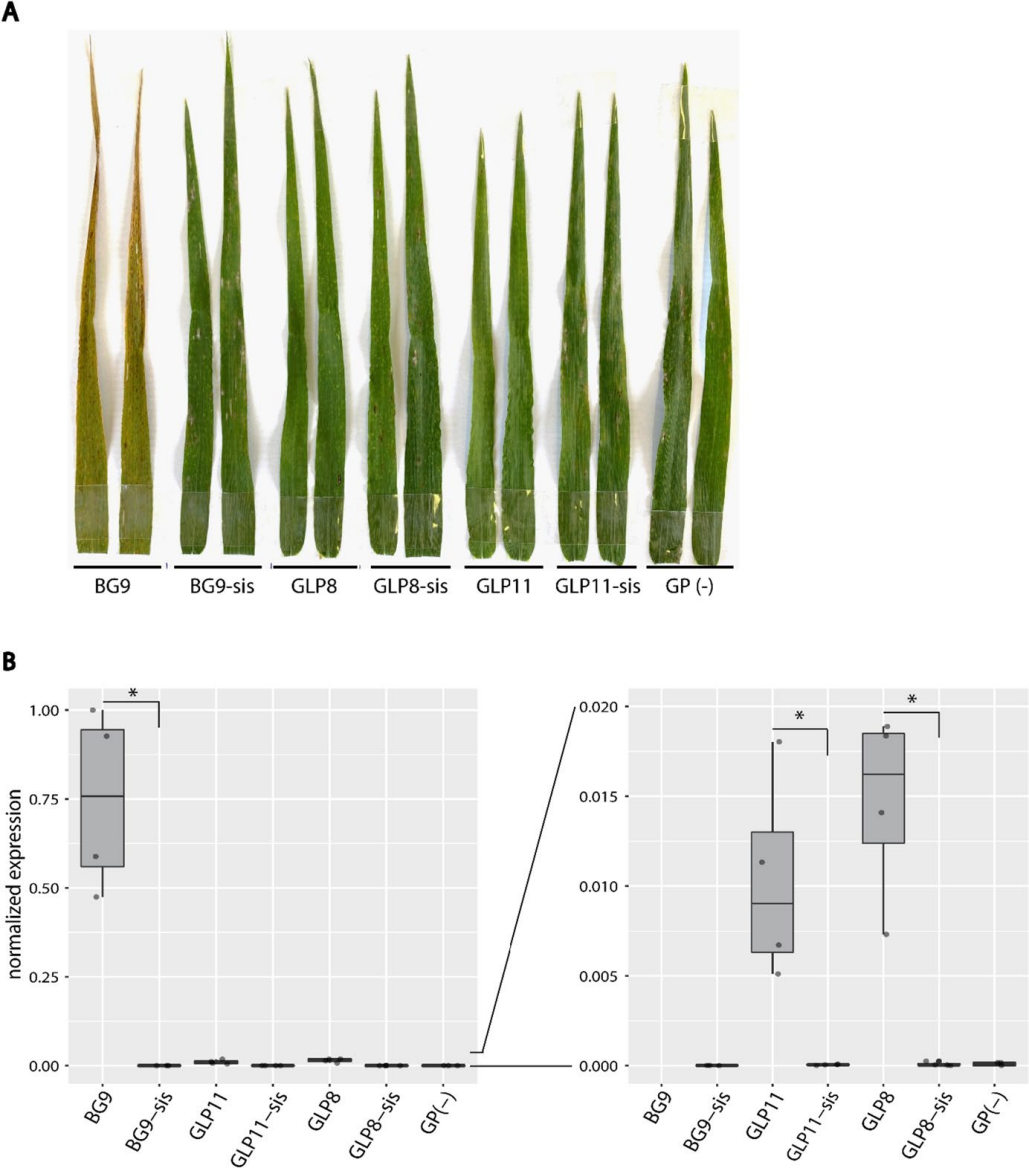
Leaf tip necrosis (LTN) and RT-qPCR assay in field-grown barley of field season 2023. **A** Photograph of barley penultimate leaves. For each line, two leaves from two different plant individuals are shown. **B** *Lr34* expression relative to reference gene ADP ribosylation factor (*ADPRF*) in field-grown barley penultimate leaves. The asterisks (*) indicate *p* < 0.05 calculated by Student’s *t*-test

Barley powdery mildew resistance test plots were artificially infected with *Bgh* isolate K1. In all tested field seasons, all three transgenic barley lines BG9, GLP8, and GLP11 were significantly more resistant against barley powdery mildew compared to their corresponding sister lines, as well as compared to untransformed GP (Fig. 3B). Furthermore, we scored flowering dates of all field-grown barley lines to detect possible pleiotropic effects of the *Lr34* transgene. In all three field seasons, there was no difference in flowering time between the transgenic- and sister lines, except for GLP11 which flowered significantly later than all other lines (Fig. 3C). In field season 2023, we additionally grew GLP8 and GLP11 together with their corresponding sister lines and GP in fungicide treated yield plots. GLP8 showed only a slight reduction in yield compared to GLP8-sis and GP; however, GLP11 showed a significant reduction in yield (Suppl. Fig. S2A). Likewise, the 1000-grain weight was very similar among the tested lines, except for GLP11 which had a significantly reduced 1000-grain weight (Suppl. Fig. S2B). This coincides with the delayed flowering of GLP11, which likely led to a shortened grain-filling period and therefore reduced yield. Another pleiotropic effect observed in the field was leaf tip necrosis (LTN) visible at the adult stage in BG9 but not in any of the other transgenic lines (Fig. 4A). Moreover, in field season 2023, we collected penultimate leaf samples from all field-grown barley lines to determine the expression of the *Lr34* transgene by an RT-qPCR assay. Line BG9 showed by far the highest *Lr34* expression of all transgenic lines, with approximately 100-fold higher expression levels than GLP8 and GLP11 (Fig. 4B). This coincided with previous studies describing stronger *Lr34* expression when expressed under the native wheat promoter than under the pathogen-inducible *Hv-Ger4c* promoter under greenhouse conditions (Boni et al. 2018) and under field conditions (Bräunlich et al. 2021). From all these observations, we concluded that *Lr34* provided resistance to two different pathogens, *Bgh* and *P. hordei*, in barley during three field seasons, however, with varying strength and sometimes pleiotropic effects. Importantly, transgenic barley line GLP8 showed partial resistance to both pathogens without any negative pleiotropic effects.

## Discussion

### High breeding value of *Pm3e*

Transgenic line Pm3e#2 showed complete powdery mildew resistance during five field seasons (Fig. 1A). Conversely, the corresponding sister line Pm3e#2-sis and untransformed Bobwhite were completely powdery mildew susceptible. Thus, resistance was only provided by the *Pm3e* transgene. This complements previously conducted field trials, where Pm3e#2 already provided strong powdery mildew resistance during four field seasons (2015–2018) (Koller et al. 2019). Taken together, these data over 9 years from consecutive field seasons demonstrate that overexpressed *Pm3e* confers strong and long-term powdery mildew resistance in the field. Even though no breakdown of *Pm3e-*provided resistance was observed during nine field seasons, it is not unlikely to happen in the future if deployed at large-scale, as has been shown for other major resistance genes such as *Pm8* (Bennett 1984; Kunz et al. 2023) and *Sr31* (Pretorius et al. 2000; Singh et al. 2011). Therefore, combining *Pm3e* with other resistance genes to increase durability must be considered for the sustainable use of this promising allele in wheat breeding. Furthermore, our study showed no negative impact of overexpressed *Pm3e* on yield potential or 1000-grain weight (Fig. 1B and C), showing that wheat cultivar Bobwhite is a suitable background for *Pm3e* transgene expression. However, Bobwhite is an agronomically rather uninteresting cultivar, so we tested the possibility of introducing *Pm3e* into an elite wheat cultivar to improve its powdery mildew resistance. We crossed *Pm3e* into elite spring wheat cultivar Fiorina, which only shows intermediate powdery mildew resistance, and showed that *Pm3e-*Fiorina BC3F2—seedlings were completely resistant to various powdery mildew isolates virulent on Fiorina (Fig. 1D). Our seedlings still segregated for *Pm3e*, and more rounds of self-fertilization and genotyping would be needed to obtain stably homozygous *Pm3e-*Fiorina plants for seed multiplication and subsequent field trials. Nonetheless, our results demonstrated the successful broad-spectrum resistance action of *Pm3e* in the background of elite cultivar Fiorina. Compatibility with the genetic background is essential for correct transgene function and the absence of pleiotropic effects. This has, for instance, been demonstrated in pepper, where efficiency of the major resistance genes *Me1* and *Me3*, conferring resistance to nematodes, was affected by the genetic background into which they were introduced (Barbary et al. 2014). Another example was shown for the late blight resistance gene *RB*, which was more efficient when introduced into potato cultivar Kufri Jyoti than in cultivar Kufri Bahar (Shandil et al. 2017). Alongside the field results presented in this study, *Pm3e* shows high potential in providing strong powdery mildew resistance without any negative pleiotropic effects, making it a promising allele for (elite) wheat breeding programs.

### Benefits and limitations of pyramiding four overexpressed *Pm3* alleles

Pyramiding of resistance genes has frequently been used in breeding to improve durability and increase the spectrum of resistance. Koller et al. (2018) showed that pyramiding of two *Pm3* alleles resulted in strong powdery mildew resistance in the field. Here, we showed that combining the four *Pm3* alleles *Pm3a*, *Pm3b*, *Pm3d*, and *Pm3f* resulted in complete powdery mildew resistance during five field seasons (Fig. 2A). Transgene expression analyses further revealed a tendency towards higher total *Pm3* expression in pyramided line Pm3a,b,d,f compared to the single transgenic lines (Fig. 2B). Since the primers used for our expression analysis did not distinguish between the different *Pm3* alleles, we cannot infer how much each *Pm3* allele contributed to the total *Pm3* expression in pyramided line Pm3a,b,d,f. However, genotyping confirmed the presence of all four *Pm3* alleles in Pm3a,b,d,f. Complete powdery mildew field resistance and similar protein accumulation of both Pm3a,b,d,f and Pm3f could suggest that only allele *Pm3f* is active and conferring resistance in the pyramided line, or that Pm3f is the dominant allele fully explaining the phenotype. Our expression data, however, showed higher, although not significantly, total *Pm3* expression in line Pm3a,b,d,f compared to line Pm3f. Importantly, the reduction of the Pm3f chlorotic leaf phenotype in the pyramided line suggests that the other *Pm3* alleles contribute to the phenotype and are not simply silenced. Transgene silencing and suppression are phenomena which have been observed before when combining different transgenes or alleles and should be considered when breeding pyramided lines (Rajput et al. 2022; Zorrilla-López et al. 2013; Hurni et al. 2014; Stirnweis et al. 2014; Koller et al. 2023). For resistance gene *Pm3* particularly, functional suppression among different *Pm3* alleles was previously shown in seedling infection tests when combined in double *Pm3*-transgenic wheat lines (Stirnweis et al. 2014). The suppression effect, which occurred at the post-translational level, depended on the combination of the two *Pm3* alleles and on the specific powdery mildew isolate that was used for infection. However, in field trials with the double *Pm3*-transgenic lines, two of which are the parental lines of our pyramided line Pm3a,b,d,f, no suppression was observed (Koller et al. 2018). We observed an example of transgene silencing when pyramiding powdery mildew resistance genes *Pm17* and *Pm3CS* in the same background of wheat cultivar Bobwhite. In this case, *ubi* promoter silencing likely led to the transcriptional silencing of both transgenes and thus powdery mildew susceptibility of the pyramided line (Koller et al. 2023). While it has been suggested that using the same promoter repeatedly can induce homology-dependent gene silencing, there are also many reports of transgenic plants containing several transgenes under the same promoter without any silencing effects (Zorrilla-López et al. 2013; Koller et al. 2023). In the previously mentioned field trials, Koller et al. (2018) attributed the enhanced powdery mildew resistance of the double *Pm3*-transgenic lines compared to the single transgenic lines to additive transgene expression and allele-specific combinations. We thus speculate that this also applies to pyramided line Pm3a,b,d,f, broadening the resistance spectrum and possibly increasing the durability of resistance. Furthermore, pyramiding diminished the pleiotropic phenotype of line Pm3f, restoring plant development in Pm3a,b,d,f to that of untransformed Bobwhite. Altogether, a pyramiding of four overexpressed *Pm3* alleles proved to be a successful approach for providing complete powdery mildew resistance in the field during five field seasons. To determine if this resistance is durable, field studies in many different environments as well as over long time, ultimately in a released variety, would be needed (Johnson 1984). Given the current situation for GMO plant breeding and growth in Europe, this is not feasible and can only be realized if regulations would be simplified.

### Fine-tuning of *Lr34* expression allows for disease resistance with reduced pleiotropic effects in barley

Previously, *Lr34*-transgenic barley lines BG9 and GLP8 were tested in a small trial during one field season (Bräunlich et al. 2021). In that study, BG9 showed complete resistance to barley powdery mildew and barley leaf rust as opposed to its sister line, however, accompanied by a strong LTN phenotype. We confirmed this complete resistance phenotype of BG9 during three additional field seasons and also observed strong LTN (Figs. 3A, B and 4A). Line GLP8 showed partial resistance to both pathogens in our field trials, except in field season 2023, where the same level of leaf rust disease as its corresponding sister line was observed. Further, we showed that *Lr34* in transgenic line GLP11 provided almost complete resistance to *P. hordei* and partial resistance to *Bgh* during three field seasons. Neither GLP11 nor GLP8 displayed LTN in the field. As demonstrated previously in greenhouse-grown wheat and barley, strong *Lr34* expression is associated with LTN and correlates with upregulation of senescence marker genes and several pathogenesis-related (PR) genes (Krattinger et al. 2009; Risk et al. 2012, 2013; Chauhan et al. 2015). Consistently, our expression analysis showed the strongest *Lr34* expression in line BG9 (Fig. 4B). While upregulation of PR genes in *Lr34*-expressing wheat occurs after rust infection (Hulbert et al. 2007), PR gene upregulation in transgenic barley expressing *Lr34* under the native wheat promoter is constitutive (Chauhan et al. 2015). The accumulation of abscisic acid (ABA) at the leaf tip in *Lr34*-barley seems to be involved in the induction of PR genes (Bräunlich et al. 2021). In contrast to wheat, *Lr34* under the native wheat promoter is constitutively expressed in barley already at the seedling stage leading to an early LTN phenotype and reduced yield components as was shown for line BG9 in the greenhouse (Risk et al. 2013). To reduce the pleiotropic effects of native *Lr34* expression in barley, lines GLP8 and GLP11 were generated which express *Lr34* under the pathogen-inducible promoter *Hv-Ger4c* at a lower level than BG9 (Boni et al. 2018). Indeed, lines GLP8 and GLP11 showed higher total grain weight compared to BG9 under greenhouse conditions; however, under near-field conditions, GLP11 showed reduced yield parameters. When tested under field conditions, line BG9 performed rather poorly with visible LTN and significantly reduced 1000-grain weight (Bräunlich et al. 2021); hence, we did not measure yield parameters for BG9 in our field trial. We showed that the 1000-grain weight of GLP11 but not GLP8 was significantly reduced under field conditions compared to the untransformed cultivar Golden Promise (Suppl. Fig. S2B). Since sister line GLP11-sis (null segregant) showed no reduction, we conclude that this is most likely a pleiotropic effect of the *Lr34* transgene in GLP11 and not any tissue culture effect. The total yield of line GLP8 was similar to that of its sister line GLP8-sis in the field, but line GLP11 showed a slight reduction compared to GLP11-sis. This is in accordance with previous results obtained under greenhouse conditions (Boni et al. 2018). Since the pleiotropic effects of *Lr34* were found to be expression level-dependent in barley (Chauhan et al. 2015), it is crucial to fine-tune *Lr34* expression to reduce such negative effects while still maintaining strong disease resistance. Line GLP8 demonstrated a successful first attempt at using a pathogen-inducible promoter leading to lower *Lr34* expression, no LTN, improved yield parameters, and nonetheless partial resistance to *P. hordei* and *Bgh* in the field. Similarly, a *Lr34*-expressing rice transgenic line showed lower *Lr34* expression and reduced LTN compared to other transgenic lines, yet still showed partial resistance to the rice blast fungus *Magnaporthe oryzae* (Krattinger et al. 2016). Moreover, it was also shown in *Lr34*-expressing durum wheat that LTN is not required for disease resistance (Rinaldo et al. 2017). Thus, it would be necessary to generate a large number of transgenic barley lines, using different and tissue-specific inducible promoters, to achieve optimal *Lr34* expression, which still confers resistance but with no or minimal pleiotropic effects. This highlights the importance of analyzing and adjusting transgene expression levels, especially when transferring genes with large physiological effects such as *Lr34* and *Lr67* (Chauhan et al. 2015; Milne et al. 2019). It is also likely that in a breeding program with large genetic diversity, genetic backgrounds with some compensatory activity for the pleiotropic effects of *Lr34* can be identified.

## Conclusion

### Importance of field trials and future considerations for breeding crops with durable disease resistance

Besides disease resistance, the agronomic performance in the field is of central interest for crop breeders. Therefore, it is indispensable to test newly generated lines also for plant fitness in the field setting and not only in the greenhouse since certain pleiotropic effects are only visible in the field. This was for example shown for two other transgenic wheat lines overexpressing *Pm3b*, which showed chlorotic leaves only when grown in the field but not in the greenhouse (Brunner et al. 2011), similar to line Pm3f used in this study. We observed a reduction of the chlorotic leaf phenotype in Pm3a,b,d,f, and we did not detect any pleiotropic effects negatively impacting yield in line Pm3e#2. In two *Lr34*-barley lines, we observed different pleiotropic effects, such as strong LTN in line BG9, delayed flowering, and lower yield in line GLP11. These negative effects, however, could most likely be reduced by fine-tuning expression levels of *Lr34* to achieve disease resistance without pleiotropic effects as demonstrated in line GLP8.

The genetic backgrounds of the transgenic lines in this study were cultivars Bobwhite for wheat and Golden Promise for barley, chosen based on their high transformation efficiency (Pellegrineschi et al. 2002; Schreiber et al. 2020). However, easily transformable plant genotypes often do not perform as well agronomically as elite cultivars and so are of little interest to breeders and farmers. Therefore, promising resistance genes tested successfully in the field, such as *Pm3e* and *Lr34* in this study, could be introduced into elite wheat and barley cultivars. We demonstrated a promising first attempt by introducing *Pm3e* into elite wheat cultivar Fiorina, which provided resistance to powdery mildew isolates which were virulent on Fiorina. Recent advances in genome editing technologies could facilitate the targeted introgression of resistance genes into different cultivars and speed up the lengthy breeding process (Pixley et al. 2022). For example, a new method called HI-Edit enables delivering the CRISPR-Cas9 genome-editing machinery components more efficiently into elite crop germplasm (Kelliher et al. 2019). Further, CRISPR-Cas9 has also been adapted for multiplexed editing, i.e., the simultaneous targeting of multiple DNA loci (Wang and Doudna 2023). This is particularly relevant for polyploid crops like wheat, which carry many redundant alleles or copies of the target gene. To take advantage of all the technological progress for improved crop breeding, however, genome-edited plants would need to become more politically and publicly accepted.

### Supplementary Information

Below is the link to the electronic supplementary material.

Supplementary file1 (DOCX 858 KB)

## Data Availability

All data supporting the findings of this study are included in the paper and its supplementary data or are available from the corresponding author on request.
