# Supplementary material for: Breeding for durable resistance against biotrophic fungal pathogens using transgenes from wheat
Source: Mol Breed. 2024 Jan 22;44(2):8. doi: 10.1007/s11032-024-01451-2 (PMC10803697; doi:10.1007/s11032-024-01451-2)
Supplement: Supplementary file 1 — Supplementary file1 (DOCX 858 KB) [file 11032_2024_1451_MOESM1_ESM.docx]

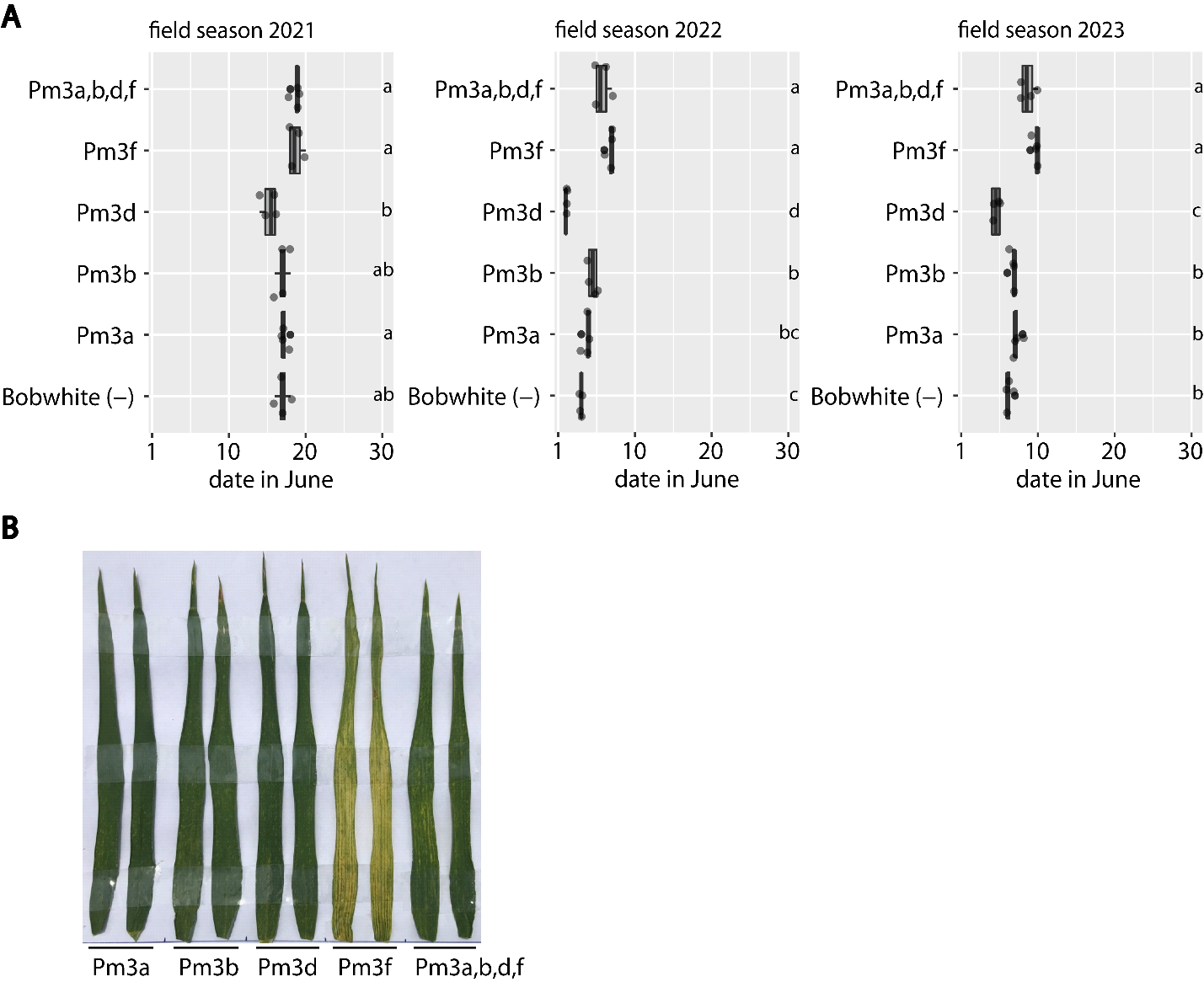


**Supplementary Fig. S1** Line Pm3a,b,d,f shows slight delayed flowering phenotype of *Pm3f* but reduction of the chlorotic leaf phenotype. A) Flowering dates of field grown transgenic wheat lines. Differing letters indicate significant difference in flowering time (Tukey HSD test, α=0.050). B) Photograph of field grown transgenic wheat flag leaves of field season 2021. For each line two leaves from two different plant individuals are shown


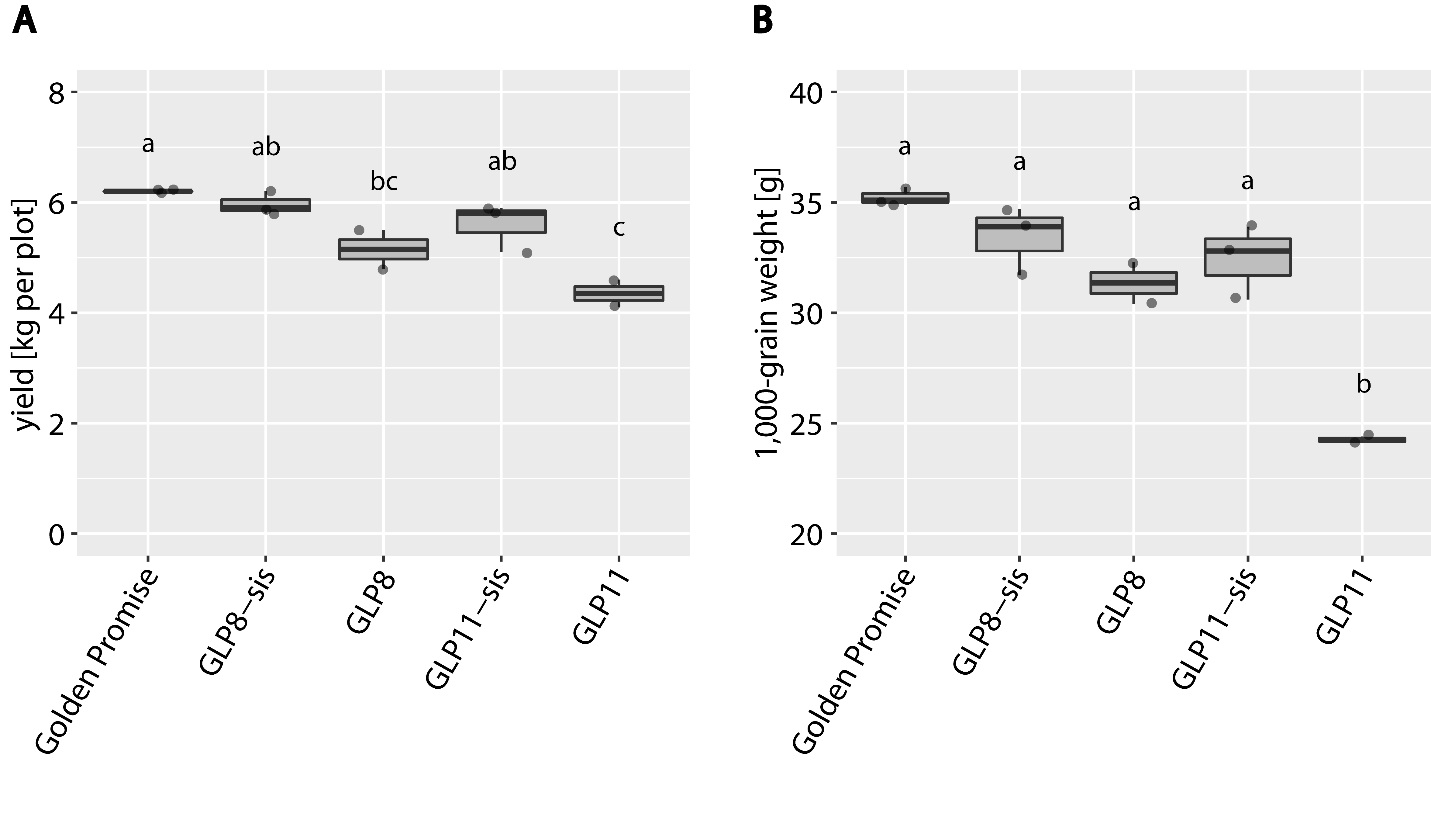


**Supplementary Fig. S2** Transgenic barley yield components in field season 2023. A) Yield per plot and B) 1,000-grain weight of field grown transgenic barley lines. Due to space limitations in the field only a few replicate plots were sown and measured (2 for GLP11 und GLP8, 3 for Golden Promise (GP), GLP8-sis and GLP11-sis). Letters above bars indicate significant difference in yield or 1,000-grain weight (TukeyHSD test, α=0.050)
